# Occurrence of Pesticide Residues in Spanish Honey Measured by QuEChERS Method Followed by Liquid and Gas Chromatography–Tandem Mass Spectrometry

**DOI:** 10.3390/foods10102262

**Published:** 2021-09-24

**Authors:** Roberto Jesús Lasheras, Regina Lázaro, Juan Carlos Burillo, Susana Bayarri

**Affiliations:** 1 Laboratorio Agroambiental, Unidad Técnica de Residuos Fitosanitarios, Gobierno de Aragón, Avenida de Montañana 1005, 50071 Zaragoza, Spain; rjlasheras@aragon.es (R.J.L.); jcburillo@aragon.es (J.C.B.); 2 Instituto Agroalimentario de Aragón—IA2, Veterinary Faculty, Universidad de Zaragoza—CITA, 50013 Zaragoza, Spain; sbayarri@unizar.es

**Keywords:** environmental pollution, pesticides, biomonitoring, honey, QuEChERS, risk assessment, apiculture

## Abstract

In the current study, the QuEChERS extraction method with slight modifications, followed by liquid and gas chromatography–tandem mass spectrometry, was applied for the determination of 399 pesticide residues in 91 raw honey samples from northeastern Spain. The quality control procedure established in Document No. SANTE/12682/2019 was successfully followed: the responses in reagent blank and blank honey samples were below 30% of the reporting limit (0.01 mg kg^−1^) for all analysed compounds, the correlation coefficients (R^2^) were higher than 0.99 in most calibration curves, the deviation of back-calculated concentration from the true concentration was below ±20% (using the standard of 50 μg L^−1^ concentration), and the recoveries of spiked samples on matrix were within the range of 70–120% for almost all analytes. Only chlorfenvinphos (2–7.8 ng/g) and coumaphos (8.8–37 ng/g) were detected in 13 samples, and neither were observed to exceed their maximum residue limits (MRLs). Dietary risk assessment for pesticide residues in honey above their lowest calibrated level (LCL) was performed, and two different age groups, adults and infants, were considered as populations at risk. The contribution of honey lay far below the acceptable daily intake (ADI) for both pesticide residues. Therefore, according to our results, honey is unlikely to pose concerns for consumer health in terms of its contribution to dietary long-term exposure. However, to maintain the level of compliance, pesticide residues in honey should be continuously monitored.

## 1. Introduction

It is widely acknowledged that bees play a major function in the environment; products derived from bees play a positive role in the global economy. According to the European Commission, it is estimated that pollinators, including honey bees, bumblebees, and wild bees, contribute at least EUR 22 billion (EUR 22,000,000,000) each year to the European agriculture industry. They ensure pollination for over 80% of crops and wild plants in Europe. Bee health depends on many factors and is endangered by changing environmental conditions, such as habitat loss, climate change, invasive species, and pesticide use [1].

The contamination sources capable of affecting bees and bee products can be roughly divided into environmental and apicultural ones. In fact, pesticide residues used in agriculture can reach the raw materials of bee products (nectar, honeydew, pollen, plant exudates) by way of air, water, plants, and soil, after which they can be transported into the beehive by the bees [2].

However, there is a considerable amount of evidence to the contrary, and the effect of pesticide residues on bees is a well-known example [3]. Recently, EFSA issued a peer review of the pesticide risk assessment for bees of three neonicotinoid insecticides (imidacloprid, clothianidin and thiamethoxam), widely used worldwide in corn, rapeseed, cotton, and sunflower crops. They confirmed the risk of their use, except in the case of permanent greenhouses. The European Union subsequently banned their outdoor use [4,5].

Therefore, bees and bee products can provide substantial information on the extent of pesticide usage in the field crops surrounding beehives; in this way, they can also act as an excellent sentinel for monitoring contamination in the environment [6].

Additionally, direct contamination of honey and other bee products can originate from beekeeping practices themselves. The most important contaminants are the residues of substances used to control bee pests, especially acaricides used against varroosis, the parasitic infestation caused by *Varroa destructor* Anderson & Trueman, which, in turn, has the largest negative economic impact on beekeeping. The lipophilic active ingredients of acaricides used in apiculture are very stable; they can accumulate in the wax comb and can contaminate honey by diffusion of the active ingredient from contaminated beeswax [7].

The presence of residues of pesticides and veterinary drugs in honey (the latter used in agriculture and apiculture to increase production, to treat infections, or for prophylactic reasons) is regulated by the European Union [8].

The determination of pesticide residues in food requires sample preparation (extraction, purification, concentration) and analytical determination (identification, separation, and quantification). All these steps are necessary due to distinct chemical properties, the complexity of matrices, and the low concentration of pesticides in food samples.

The sample preparation step is of fundamental importance. Traditional sample preparation methods (liquid–liquid extraction, Soxhlet extraction, etc.) are laborious, time consuming, expensive, require large amounts of organic solvents, and usually involve many steps. These limitations have led to the development of new techniques that are convenient, consume less organic solvents, and have the ability to detect analytes in very low concentrations, such as accelerated solvent extraction (ASE), supercritical fluid extraction (SFE), microwave-assisted extraction (MAE), solid phase extraction (SPE), solid phase microextraction (SPME), matrix solid phase dispersion (MSPD), and extraction combined with QuEChERS (quick, easy, cheap, effective, rugged, and safe) [9].

The QuEChERS technique was developed in 2003 for the determination of multiclass pesticide residues in fruits and vegetables [10] and subsequently modified to improve recoveries of pH-dependant analytes by using buffering salts during the extraction step. Finally, the European Standard EN document No. 15662, entitled “Foods of Plant Origin—Determination of Pesticide Residues Using GC–MS and/or LC–MS/MS Following Acetonitrile Extraction/Partitioning and Clean-up by Dispersive SPE (QuEChERS method)”, was published in 2008 and revised in 2018 [11]. In recent years, this technique has been applied to the analysis of pesticide residues in honey [6,12,13,14,15,16].

The present study includes multiple approaches to the investigation of pesticide residues in honey. The aim was to evaluate the presence of 399 pesticide residues in Spanish honeys of different botanical origins, using the modified QuEChERS method followed by determination with liquid and gas chromatography–mass spectrometry (129 and 270 compounds, respectively). The compounds were selected based on the Commission Implementing Regulation (EU) 2019/533 and have been recommended as target pesticides for proficiency tests by the European Reference Laboratories, including other relevant compounds used in apiculture (tau-fluvalinate, coumaphos, and amitraz), and further compounds toxic to bees (clothianidin, thiamethoxam, and imidacloprid) [17]. Similarly, we evaluated the contribution of honey to long-term dietary exposure and risk assessment.

## 2. Materials and Methods

### 2.1. Chemicals and Reagents

LC–MS grade acetonitrile, methanol, and water were ordered from Merck KGaA, Darmstadt, Germany for use in LC–MS. Cyclohexane and ethyl acetate for analysis EMSURE (Merck KGaA, Darmstadt, Germany) were used for GC–MS.

The QuEChERS kit that we used for the extraction step was the DisQuE Dispersive SPE Kit (CEN Method 15662) (Waters) with a 50 mL tube with 4 g MgSO_4_, 1 g NaCl, 1 g trisodium citrate dihydrate, and 0.5 g disodium hydrogen citrate sesquihydrate. For the clean-up step, we used a 15 mL tube with 900 mg of magnesium sulphate and 150 mg PSA (Primary-Secondary Amine) sorbent.

### 2.2. Analytes and Standard Solutions

Compounds investigated are shown in Tables S1 and S2. Pesticide standards were purchased from CPAChem Ltd. (Bulgaria, France). The custom pesticide mix solutions of 100 mg L^−1^ we used were as follows: one in methanol for liquid chromatography and another in cyclohexane for gas chromatography (GC). Two standard mix solutions for calibration were prepared in acetonitrile for LC–MS and cyclohexane:ethyl acetate (90:10) at 10 mg L^−1^. All standard solutions were stored at −20 °C. Standard calibration solutions were prepared at seven concentration levels ranging from 2 to 250 µg L^−1^. A matrix-matched calibration was used to compensate matrix effects. An extract of blank honey was used for LC–MS and GC–MS calibrations for the matrix-matched standard calibration.

One analytical grade standard of triphenyl phosphate (TPP), ordered from Dr. Ehrenstorfer, Germany, was used as surrogate standard. An individual solution was prepared by dissolving the standard in methanol at a concentration of 10 mg L^−1^.

### 2.3. Honey Samples

Beekeepers filled out a form indicating date of collection and floral origin of honeys. Samples were prepared according to the Quality Control procedure established in “Analytical quality control and method validation procedures for pesticide residues analysis in food and feed” [18]. Following this document, the samples were perfectly homogenised upon their arrival at the laboratory. All samples were kept at −15 °C until analysis. Ninety-one raw honey samples from different locations in northeastern Spain were analysed. Floral origins of honey samples were confirmed by pollen analysis: 54 multifloral, 20 from *Lamiaceae* (*Rosmarinus officinalis* + *Thymus vulgaris*), 10 from blackberry (*Rubus* sp.), and 7 from fruit trees (*Prunus* + *Pyrus*). Samples were collected during the 2017–2019 period.

### 2.4. Extraction Sample

For multiresidue analysis of pesticides in honey, the QuEChERS method according to European Standard EN 15662:2018 [11] was used with slight modifications. A 5 ± 0.1 g portion of sample was weighed in a 50 mL centrifuge tube, and a 20 μL volume of 10 mg L^−1^ TPP standard in methanol was added as surrogate standard. Then, 10 mL of water and 10 mL of acetonitrile were added with 4 g MgSO_4_, 1 g NaCl, 1 g trisodium citrate dihydrate, and 0.5 g disodium hydrogencitrate sesquihydrate; the sample was shaken vigorously for 10 min. The extract was centrifuged for 5 min at 2500 rpm. In the second step (clean-up), an aliquot of the extract (6 mL) was transferred into a polypropylene single centrifuge tube that contained 600 mg magnesium sulphate and 25 mg PSA sorbent, which was shaken vigorously for 10 min and centrifuged for 5 min at 2500 rpm. Two aliquots of 1 mL of the supernatant were collected in two chromatography vials. One of them was analysed by LC–MS, and the other was evaporated to near dryness with a stream of nitrogen and reconstituted with cyclohexane:ethyl acetate (80:20) for GC–MS analysis.

### 2.5. Quality Control

The following quality control samples were analysed: reagent blank, honey sample blank, and one honey sample spiked at limit of quantification (0.01 mg kg^−1^). The specificity was evaluated using the reagent blank and blank control samples, with an acceptance criterion for their response lying below 30% of the limit of quantification. Recovery was evaluated using a spiked sample prior to extraction with all analysed compounds at the limit of quantification. An acceptable range for routine recoveries of 60–140% was applied for individual recoveries. These criteria follow Document No. SANTE/12682/2019 [18].

The samples, calibration standards, and quality control were processed and injected into the same batch.

### 2.6. Chromatographic Analysis

The residues in cleaned-up extracts were detected and quantified using liquid and gas chromatography coupled to a mass spectrometer, triple quadrupole, and liquid chromatography coupled to mass spectrometer, triple quadrupole. The compounds were identified on the basis of the following parameters: retention time (±0.1 min), a quantification transition, a qualification transition, and the ion ratio of those transitions (±30%).

One of the most relevant residue pesticides in honey is amitraz, the current definition of which is “amitraz including the metabolites containing the 2,4-dimethylaniline moiety expressed as amitraz” [19]. The two-dimethylaniline moieties are *N*-2,4-dimethylphenyl-*N*′-methyl-formamidine and *N*-2,4-dimethylphenyl-formamide. These compounds can be decomposed to 2,4-dimethylaniline by means of strong alkaline hydrolysis. However, amitraz (sum) can be calculated from *N*-2,4-dimethylphenyl-*N*′-methyl-formamidine using a 1:1 stoichiometry between *N*-2,4-dimethylphenyl-*N*′-methyl-formamidine and amitraz [20]. Therefore, amitraz and *N*-2,4-dimethylphenyl-*N*′-methyl-formamidine were monitored through LC–MS/MS.

### 2.7. GC–MS/MS

A gas chromatograph (Agilent 7890N) coupled to triple quadrupole (Agilent 7000D) was used as the GC–MS/MS system. A spitless injector (280 °C, spitless time 0.75 min) was used, and the injection volume was 2 μL. Helium (99.999%) was used as the carrier gas at a constant flow of 1 mL min^−1^. GC separation was performed on two capillary columns (DB5MS, 15 m × 0.25 mm i.d., 0.25-μm-thick film). The oven program was initially at 60 °C for 1 min, and the temperature increased to 170 °C at a rate of 40 °C min^−1^, increased to 310 °C at a rate of 10 °C min^−1^, and was held for 3 min at 310 °C. Finally, one post-run for 4 min to 310 °C in blackflush mode was used. The triple quadrupole mass spectrometer parameters were as follows: quadrupole temperature, 150 °C; MS transfer line and ion-source temperature, 280 °C; collision gas (nitrogen), 15 mL min^−1^; and quenching gas (helium), 2.25 mL min^−1^. The retention times, quantification and confirmation transitions, and collision energies for each compound are shown in Table S1.

### 2.8. LC–MS/MS

The LC–MS system used was an HPLC system coupled to an MS/MS system (Varian 320-MS) operating in electrospray ionisation mode. Chromatographic separation was performed on a reversed-phase C18 column of 150 mm × 3 mm and 3 μm particle size thermostated at 40 °C. The flow rate was 200 μL min^−1^. The mobile phases were as follows: phase A consisted of water with 0.2% formic acid and 5 mM ammonium formate, and phase B of methanol and a 10% phase A. The gradient started with 60% of B, followed by a linear gradient up to 70% in 5 min, followed by a linear gradient up to 100% of 4 min, then constant for 6 min, and 4 min of post-time using the initial 60% of B. The injection volume was 10 μL.

The electrospray ionisation (ESI) source was operated in positive and negative mode. Its parameters were as follows: nebulising gas (nitrogen), 60 psi; capillary voltage, 5000 V; shield voltage, 600 V; drying gas, 35 psi at 400 °C. Argon at 2 mTorr served as collision gas.

LC–MS/MS identification was carried out regarding the retention time and the ion ratios obtained. MS transitions, polarity, retention time, capillary voltage, and collision energy for each compound are shown in Table S2.

### 2.9. Dietary Risk Assessment

Dietary risk assessment for pesticide residues detected in honey above their LCL was performed. Two different age groups were considered as populations at risk: adults (70 kg body weight) and infants (12 kg body weight).

A comparison of estimated long-term dietary exposure with a relevant toxicological reference value for long-term exposure such as ADI provides an indication of whether consumers are exposed to pesticide residues that may pose a health risk [21]. The ADI (mg of residue kg^−1^ bw per day) is the amount of a chemical (in this case, pesticide residue) to which a person can be exposed daily over a long period without suffering harmful effects [22]. Based on current scientific knowledge, when dietary exposure to a substance is found to be lower than or equal to its health-based guidance value, the health risk for the consumer is low. When it exceeds its health-based guidance value, possible negative health outcomes cannot be excluded.

Consequently, the contribution of honey to long-term dietary exposure (EDI) was estimated by multiplying the concentrations of detected pesticide residues by the average daily per capita consumption of honey on the basis of data from the Spanish Ministerio de Agricultura, Pesca y Alimentación [23]. The value was finally divided by body weight, and results were expressed as μg pesticide kg body weight^−1^ day^−1^: EDI = L × C/Bw, where L (μg kg^−1^) is the average level of a given pesticide residue in honey samples, C (kg d^−1^) is the daily per capita consumption of honey, and Bw is the body weight (kg).

Finally, dietary risk assessment was evaluated with the HQ parameter, calculated by dividing the EDI by the ADI for each pesticide: HQ = EDI/ADI.

## 3. Results and Discussion

### 3.1. Pesticide Residues

Quality controls were successful; the responses in reagent blank and blank honey samples were below 30% of the reporting limit (0.01 mg kg^−1^) for all analysed compounds, and the correlation coefficients (R^2^) were higher than 0.99 in most calibration curves. The response drift of the determination systems was confirmed by bracketing calibration. The deviation of the back-calculated concentration from the true concentration was below ±20% when using the reinjected standard of 50 μg L^−1^ concentration at the end of a sample sequence, and the recoveries of the spiked samples on the matrix were within the acceptance range of Document No. SANTE/12682/2019 for most compounds [18].

Recoveries lower than 70% in the GC–MS/MS system were obtained for the following compounds: dicofol (degraded to 4,4-dichlobenzofenone in the injector), chlorothalonil (due to the use of PSA in the clean-up step), tolylfluanid (decomposed to DMST), captan (degraded to tetrahydrophthalimide), folpet (degraded to phthalimide), and diclofluanide. Recoveries lower than 70% in the LC–MS/MS system were obtained for the following compounds: pymetrozine, benfuracarb, clothianidin, fenbutatin oxide, spirotetramat cis enol, and spirotetramat enol glucoside.

Table 1 shows the results of pesticide residues found in the honey samples analysed in our study. Out of the 399 pesticide residues investigated, only the organophosphorus pesticides chlorfenvinphos (7 samples) and coumaphos (6 samples) were found to be above the LCL, and only four pesticide residues were detected above the LOQ. Positive samples showed only one of the two residues. Both pesticides are associated with livestock treatments, and the results showed that coumaphos residue levels (8.8–37 ng g^−1^) were higher than those of chlorfenvinphos (2–7.8 ng g^−1^). No other pesticides analysed used in agriculture or apiculture treatments and no other environmental contaminants were detected. No conclusions could be drawn about the influence of the botanical origin of the samples on the contamination pattern due to the different number of samples in each group.

Chlorfenvinphos is no longer authorised for agricultural use in the EU, nor is it authorised for treatment against *Varroa* mite in bees [24]. As no MRL is established for this substance, a default value of 0.01 mg kg^−1^ is applied for honey [8]. The presence of this residue in the samples could be associated with the use of contaminated recycled wax or with illegal treatments.

Levels of chlorfenvinphos residues obtained in this study are similar to those found in Andalusian (Spain) honeys (6 ng g^−1^) [25]. In neighbouring countries such as Italy, the reported average concentration was 19.67 ng g^−1^ [26]. On the other hand, rather low levels (0.70–0.89 ng g^−1^) were reported in Greek honeys (chlorfenvinphos, chlorpyrifos, and phorate) [27].

Coumaphos residues in honey more likely originate from treatments of beehives with antiparasitic products authorised by the European Union for veterinary medicinal products rather than from pesticide uses [19]; this compound is no longer approved as a pesticide in Europe [21]. Levels of coumaphos residues found in our study lie far below the maximum residue level (0.1 mg kg^−1^ honey). The presence of this residue in our samples could be associated with intensive use related to *Varroa* treatment, as discussed above. The conditions of use are very important. In an experimental study, after a therapeutic dose of coumaphos (CheckMite; Bayer, Berlin, Germany), and according to the manufacturer’s instructions, the results indicated the undetectable presence of coumaphos in honey (below LOQ 50 μg kg^−1^) [28].

On the other hand, other researchers analysed certified organic and conventional honeys from Croatia and found one or two synthetic acaricides in some samples, with coumaphos being most frequently detected [29]. In addition, certain recent studies carried out in Spain showed an increase in the use of this pesticide due to an increase in resistance of *V. destructor* to tau-fluvalinate [30].

Pesticide analysis of commercial honeys of polyfloral origin carried out in Spain showed amitraz (in 100% of samples) and coumaphos (in 63%, up to 14 mg kg^−1^) as the most detected residues [31]. The same residues were detected in another study on Spanish honeys from apiaries located in rural and forest areas. Coumaphos residue levels were in the range of 6–36 µg kg^−1^ [25]. Similarly, amitraz metabolites and coumaphos were the most frequently detected residues on honey samples from Israeli apiaries [32]. Residues of diazinon, mevinphos, coumaphos, chlorpyrifos, and quinoxyfen were detected on Italian organic honeys coming from apple and citrus orchard areas [33].

Regarding the multiresidue presence of pesticides in honey, another study has reported residues of tau-fluvalinate (among 200 pesticides investigated) in one group of 64 commercial honey samples of various botanical origins from Egypt, which is in accordance with our results [15]. Similarly, an analysis of 61 Colombian honey samples resulted in 32 positive samples and only 5 samples contaminated with more than 1 pesticide (3 residues in 3 samples, and 4 residues in 2 samples). Among the 53 analysed compounds, the main ones detected were chlorpyrifos, profenofos, DDT, HCB, γ-HCH, and fenitrothion [34]. More recently, 21 out of 207 analysed pesticides were found in Polish honey samples [14]. Samples containing a residue of only one pesticide accounted for 17% of the total. Three pesticide residues on average were detected simultaneously in honey samples, although more than four pesticides were also found in 17% of samples, and a maximum of nine residues were detected in one sample. Cyano-substituted neonicotinoids were the most frequently detected pesticides (77% of samples).

The 2018 European Union report on pesticide residues in food [21] analysed 1131 samples of honey and other apicultural products. Among the 619 different pesticides sought in honey and related products, 30 were quantified, the most common being copper, thiacloprid, glyphosate, chlordane, dimoxystrobin, and chlorate. Other pesticides similarly quantified in honey included coumaphos (as found in our study) as well as environmental contaminants resulting from pesticide use in the past (e.g., chlordane, DDT, methoxychlor, heptachlor, HCH alpha and beta, lindane, and dieldrin). The results showed that 236 samples (20.1%) contained at least residues from 1 pesticide, 19 samples numerically exceeded the MRL (1.7%), and 9 samples were reported as MRL non-compliant by the countries that were reporting (0.8%). The MRL was exceeded for glyphosate, amitraz, fluvalinate, and coumaphos.

In Spain, the presence of 322 chemical residues was evaluated in beeswax, bee bread, and honey by LC–MS/MS and GC–MS/MS [25]. Residues of acaricides used for sanitary treatments, coumaphos, and two transformation products of amitraz (DMF and DMPF) were quantified in honey. All, except one sample, were below the EU MRLs [8].

### 3.2. Dietary Risk Assessment

Table 2 summarises results for risk assessment. Estimated daily intakes of chlorfenvinphos and coumaphos were compared with their ADI (0.0005 and 0.00025 mg of residue kg^−1^ bw per day, respectively) [35,36]. HQ results indicate that the contribution of honey to chronic dietary exposure, for both adults and children and for both pesticides, amounted to less than 1% of the ADI, except for coumaphos when considering exposure of children (3.65%).

These results are in accordance with those provided by the 2018 European Union report on pesticide residues in food [21], which found that honey is a minor contributor to dietary exposure to pesticide residues.

Nevertheless, it is important to bear in mind that honey can also be used in the food industry for different purposes (Table 3). It is particularly notable that it may be present in infant cereal products as complementary elements to the diet of 6-month-old babies.

Similarly, the potential risks of combined exposures to multiple residues from pesticides in consumer diet should also be addressed. To this end, EFSA has recently published the results of its two pilot evaluations on the risks posed to humans by the residues of multiple pesticides in food: one of them addresses chronic effects on the thyroid system [47], and the other one evaluates acute effects on the nervous system [48]. The overall conclusion from both assessments is that, with varying degrees of certainty, consumer risk from cumulative dietary exposure is below the threshold that triggers regulatory action for all of the population groups covered. Assessments covering the effects of pesticides on other organs and body functions will follow in the coming years.

EFSA [21] recommends for honey samples to be analysed by member states under their national programmes, maintaining the analytical scope as wide as possible, including, as a minimum, the following pesticides: acetamiprid, amitraz, boscalid, dimoxystrobin, glyphosate, and thiacloprid.

## 4. Conclusions

In conclusion, we evaluated the presence of residues of 399 pesticides in 91 samples of raw honey with the goal of contributing to food safety and protecting public health. The QuEChERS method coupled with LC/MS and GC/MS, successfully validated according to European Commission requirements, was used. Chlorfenvinphos and coumaphos residues were the pesticide residues detected, and none were above their respective MRL. The long-term exposure assessment estimated that the contribution of honey was far below the ADI for both pesticide residues. Therefore, according to our results, honey is unlikely to pose concerns for consumer health regarding the role it plays in dietary long-term exposure. Continuous monitoring of pesticide residues in honey should nevertheless be carried out to maintain the level of compliance.

## Figures and Tables

**Table 1:** Pesticide residues in raw honey samples according to their botanical origin.

Botanical Origin	Number of Samples	Number of Positive Samples	Residue Detected	Residue Level (ng g^−1^ Honey)
Multifloral	54	3	Chlorfenvinphos	2.0 ± 1.0
				2.0 ± 1.0
				4.5 ± 2.3
		6	Coumaphos	26.5 ± 13.2
				9.1 ± 4.6
				12.0 ± 6.0
				37.0 ± 18.5
				8.8 ± 4.4
				15.2 ± 7.6
*Lamiaceae* (*Rosmarinus officinalis* + *Thymus vulgaris*)	20	1	Chlorfenvinphos	7.8 ± 3.9
Blackberry (*Rubus* sp.)	10	3	Chlorfenvinphos	2.0 ± 1.0
				2.0 ± 1.0
				3.9 ± 2.0
Fruit trees (*Prunus* + *Pyrus*)	7	0	-	-

**Table 2:** Risk assessment of chlorfenvinphos and coumaphos due to honey consumption.

Residue	Age Group	EDI ^1^ (μg kg^−1^ Body Weight/Day)	ADI (mg kg^−1^ Body Weight/Day)	HQ (%)
Chlorfenvinphos	Adults	3.38 × 10^−7^	0.0005 (47)	0.07
	Children	1.97 × 10^−6^		0.39
Coumaphos	Adults	1.56 × 10^−6^	0.00025 (44)	0.62
	Children	9.13 × 10^−6^		3.65

^1^ EDI has been estimated assuming a daily honey consumption of 0.4 kg per person per year (Ministerio de Agricultura, Pesca y Alimentación, 2020), and a body weight 70 kg for adults and 12 kg for children. When the detected residues in the sample were below the LOQ, values were treated as 0.

**Table 3:** Main applications of honey in the food industry.

Product	Applications	Featured References
Cereals and cereal-based products	Sweetener	[37,38]
	Nutrient ingredient	
	Adhesive material	
	Binding agent	
	Improves colour, aroma, and viscosity	
	Improves darkening	
	Improves shelf life	
Bakery and pastry	Increases water retentionImproves colour, aroma, and texture	[39]
	Improves darkening	
	Improves shelf life	
	Good mouthfeel in reduced fat products	
	Sweetener	
	Functional ingredient	
Sweet	Sweetener	[40]
	Topping	
	Binding agent	
	Improves viscosity and texture flavouring	
Meat and meat products	Increases water retention	[41,42,43]
	Improves colour	
	Improves browning	
	Reduces the formation of heterocyclic aromatic amines and their mutagenic effectsFlavouring	
	Good mouthfeel in reduced fat products	
Dairy products and ice creams	Natural preservative	[44,45]
	Sweetener	
	Ingredient prebiotic	
	Reduces freezing point	
	Flavouring	
Beverages	Sweetener	[46]
	Clarifying	
	Colour enhancement	
	Flavouring	

## Supplementary Materials

The following are available online at https://www.mdpi.com/article/10.3390/foods10102262/s1, Table S1: List of Compounds Included in the GC–MS/MS method together with their Retention Times (RT), quantification and confirmation transitions, and collision energies. Table S2: List of Compounds Included in the LC–MS/MS method together with their Retention Times (RT), quantification and confirmation transitions, capillary voltage, and collision energies.

## References

[1] European Comission Bees Honey. https://ec.europa.eu/food/animals/live_animals/bees_en.

[2] Bogdanov S. (2006). Contaminants of bee products. Apidologie.

[3] Nicolopoulou-Stamatti P., Malpas S., Kotampasi C., Stamatis P., Hens L. (2016). Chemical pesticides and human health: The urgent need for a new concept in agriculture. Front. Public Health.

[4] EFSA (European Food Safety Authority) (2018). Conclusion on the peer review of the pesticide risk assessment for bees for the active substance clothianidin considering the uses as seed treatments and granules. EFSA J..

[5] EFSA (European Food Safety Authority) (2018). Conclusions on the peer review of the pesticide risk assessment for bees for the active substance thiamethoxam considering the uses as seed treatments and granules. EFSA J..

[6] Kumar A., Gill J.P.S., Bedi J.S., Kumar A. (2018). Pesticide residues in Indian raw honeys, an indicator of environmental pollution. Environ. Sci. Pollut. Res..

[7] Adamczyk S., Lázaro R., Pérez-Arquillué C., Herrera A. (2007). Determination of synthetic acaricides residues in beeswax by high-performance liquid chromatography with photodiode array detector. Anal. Chim. Acta.

[8] European Comission (2005). Regulation (EC) No 396/2005 of the European Parliament and of the Council of 23 February 2005 on Maximum Residue Levels of Pesticides in or on Food and Feed of Plant and Animal Origin and Amending Council Directive 91/414/EEC.

[9] Ðurović R., Ðorđević T. (2011). Modern extraction techniques for pesticide residues determination in plant and soil samples. Pesticides in the Modern World—Trends in Pesticides Analysis.

[10] Anastassiades M., Lehotay S.J., Štajnbaher D., Schenck F.J. (2003). Fast and easy multiresidue method employing acetonitrile extraction/partitioning and ‘dispersive solid-phase extraction’ for the determination of pesticide residues in produce. J. AOAC Int..

[11] European Committee for Standardization (2018). Foods of Plant Origin—Multimethod for the Determination of Pesticide Residues Using GC-and LC-Based Analysis Following Acetonitrile Extraction/Partitioning and Clean-Up by Dispersive SPE—Modular QuEChERS-Method.

[12] Niell S., Jesus F., Perez C., Mendoza Y., Diaz R., Franco J., Cesio V., Heinzen H. (2015). QuEChERS adaptability for the analysis of pesticide residues in beehive products seeking the development of an agroecosystem sustainability monitor. J. Agric. Food Chem..

[13] Chiesa L.M., Labella G.F., Panseri S., Britti D., Galbiati F., Villa R., Arioli F. (2017). Accelerated solvent extraction by using an ‘in-line’clean-up approach for multiresidue analysis of pesticides in organic honey. Food Addit. Contam. Part A.

[14] Gaweł M., Kiljanek T., Niewiadowska S., Semeniuk A., Goliszek M., Burek O., Posyniak A. (2019). Determination of neonicotinoids and 199 other pesticide residues in honey by liquid and gas chromatography coupled with tandem mass spectrometry. Food Chem..

[15] Shendy A.H., Al-Ghobashy M.A., Mohammed M.N., Alla S.A.G., Lotfy H.M. (2016). Simultaneous determination of 200 pesticide residues in honey using gas chromatography-tandem mass spectrometry in conjunction with streamlined quantification approach. J. Chromatogr. A.

[16] Zamudio A.M., Vanoy N., Díaz-Moreno C., Ahumada D.A. (2017). Development and validation of a multiresidue method for pesticide analysis in honey by UFLC-MS. Rev. Colomb. Química.

[17] European Comission (2019). Commission Implementing Regulation (EU) 2019/533 of 28 March 2019 Concerning a Coordinated Multiannual Control Programme of the Union for 2020, 2021 and 2022 to Ensure Compliance with Maximum Residue Levels of Pesticides and to Assess the Consumer Exposure.

[18] European Commission DG-SANTE (2019). Analytical Quality Control and Method Validation Procedures for Pesticide Residues Analysis in Food and Feed.

[19] European Comission (2010). Commission Regulation (EU) No 37/2010 of 22 December 2009 on Pharmacologically Active Substances and Their Classification Regarding Maximum Residue Limits in Foodstuffs of Animal Origin.

[20] Hepperle J., Mack D., Sigalov I., Schüler S., Anastassiades M. (2015). Analysis of ‘amitraz (sum)’ in pears with incurred residues—Comparison of the approach covering the individual metabolites via LC-MS/MS with the approach involving cleavage to 2,4-dimethylaniline. Food Chem..

[21] Medina-Pastor P., Triacchini G., EFSA (European Food Safety Authority) (2020). The 2018 European Union report on pesticide residues in food. EFSA J..

[22] World Health Organization, Inter-Organization Programme for the Sound Management of Chemicals (2004). IPCS Risk Assessment Terminology. http://www.inchem.org/documents/harmproj/harmproj/harmproj1.pdf.

[23] Ministerio de Agricultura Pesca y Alimentación (2020). Informe del Consumo de Alimentación en España 2019. https://www.mapa.gob.es/es/alimentacion/temas/consumo-tendencias/informe2019_v2_tcm30-540250.pdf.

[24] European Comission (2002). Commission Regulation (EC) No 2076/2002 of 20 November 2002 Extending the Time Period Referred to in Article 8(2) of Council Directive 91/414/EEC and Concerning the Non-Inclusion of Certain Active Substances in Annex I to that Directive and the Withdrawal.

[25] Lozano A., Hernando M.D., Uclés S., Hakme E., Fernández-Alba A.R. (2019). Identification and measurement of veterinary drug residues in beehive products. Food Chem..

[26] Saitta M., Di Bella G., Fede M.R., Lo Turco V., Potortì A.G., Rando R., Russo M.T., Dugo G. (2017). Gas chromatography-tandem mass spectrometry multi-residual analysis of contaminants in Italian honey samples. Food Addit. Contam. Part A.

[27] Balayiannis G., Balayiannis P. (2008). Bee honey as an environmental bioindicator of pesticides’ occurrence in six agricultural areas of Greece. Arch. Environ. Contam. Toxicol..

[28] Bajuk B.P., Babnik K., Snoj T., Milčinski L., Ocepek M.P., Škof M., Jenčič V., Filazi A., Štajnbaher D., Kobal S. (2017). Coumaphos residues in honey, bee brood, and beeswax after Varroa treatment. Apidologie.

[29] Lazarus M., Lovaković B.T., Orct T., Sekovanić A., Bilandžić N., Đokić M., Kolanović B., Varenina I., Jurič A., Lugomer M.D. (2021). Difference in pesticides, trace metal(loid)s and drug residues between certified organic and conventional honeys from Croatia. Chemosphere.

[30] Alonso-Prados E., Muñoz I., De la Rúa P., Serrano J., Fernández-Alba A.R., García-Valcárcel A.I., Hernando M.D., Alonso Á., Alonso-Prados J., Bartolomé C. (2020). The toxic unit approach as a risk indicator in honey bees surveillance programmes: A case of study in Apis mellifera iberiensis. Sci. Total Environ..

[31] Juan-Borrás M., Domenech E., Escriche I. (2016). Mixture-risk-assessment of pesticide residues in retail polyfloral honey. Food Control.

[32] Bommuraj V., Chen Y., Klein H., Sperling R., Barel S., Shimshoni J.A. (2019). Pesticide and trace element residues in honey and beeswax combs from Israel in association with human risk assessment and honey adulteration. Food Chem..

[33] Chiesa L.M., Labella G.F., Giorgi A., Panseri S., Pavlovic R., Bonacci S., Arioli F. (2016). The occurrence of pesticides and persistent organic pollutants in Italian organic honeys from different productive areas in relation to potential environmental pollution. Chemosphere.

[34] López D.R., Ahumada D.A., Díaz A.C., Guerrero J.A. (2014). Evaluation of pesticide residues in honey from different geographic regions of Colombia. Food Control.

[35] Food and Agriculture Organization of the United Nations, World Health Organization (1994). FAO Panel of Experts on Pesticide Residues in Food and the Environment; WHO Expert Group on Pesticide Residues. Pesticide residues in food: 1994. Proceedings of the Joint Meeting of the FAO Panel of Experts on Pesticide Residues in Food and the Environment and the WHO Expert Group on Pesticide Residues, Rome, Italy, 19–28 September 1994.

[36] EFSA (European Food Safety Authority) (2016). Setting of maximum residue levels for amitraz, coumaphos, flumequine, oxytetracycline, permethrin and streptomycin in certain products of animal origin. EFSA J..

[37] Agbaje R., Hassan C.Z., Norlelawati A., Rahman A.A., Huda-Faujan N. (2016). Development and physico-chemical analysis of granola formulated with puffed glutinous rice and selected dried Sunnah foods. Int. Food Res. J..

[38] Kaur R., Ahluwalia P., Sachdev P.A., Kaur A. (2018). Development of gluten-free cereal bar for gluten intolerant population by using quinoa as major ingredient. J. Food Sci. Technol..

[39] Arvanitoyannis I.S., Bosinas K.P., Bouletis A.D., Gkagtzis D.C., Hadjichristodoulou C., Papaloucas C. (2011). Study of the effect of atmosphere modification in conjunction with honey on the extent of shelf life of Greek bakery delicacy ‘touloumpaki’. Anaerobe.

[40] Anwar N.Z.R., Hashim N.A., Zulkifli N.A. (2018). Melting Characteristics of Milk Chocolate with Different Sweetener Blends. Int. J. Eng. Technol..

[41] Hashim I.B., McWatters K.H., Hung Y.C. (1999). Marination method and honey level affect physical and sensory characteristics of roasted chicken. J. Food Sci..

[42] Karo F.Y., Hidayati P.I., Krisnaningsih A.T.N. (2017). The effect of honey as natural preservative to quality of meat with temperature variation on the save 4 °C. J. Sains Peternak..

[43] López-Velasco D.S., Sosa-Montes E., González-Cerón F., Pró-Martínez A., Vargas-Galicia A.J. (2021). Efecto antioxidante de la miel de abeja sobre la carne de conejo almacenada en refrigeración. CienciaUAT.

[44] Stijepic M.J., Glusac J.R., Milosevic-Djurdjevic D.M. (2009). Prebiotic effect of honey addition on fermentation and physico-chemical properties of probiotic drink produced from goat milk/Probioticko djelovanje meda na fermentaciju i svojstva kozjeg i kravljeg probiotickog jogurta. Prehrambena Ind. (Serbia).

[45] Moriano M.E., Alamprese C. (2017). Honey, trehalose and erythritol as sucrose-alternative sweeteners for artisanal ice cream. A pilot study. LWT—Food Sci. Technol..

[46] Caldeira I., Lopes D., Delgado T., Canas S., Anjos O. (2018). Development of blueberry liquor: Influence of distillate, sweetener and fruit quantity. J. Sci. Food Agric..

[47] Craig P.S., Dujardin B., Hart A., Hernandez-Jerez A.F., Hougaard Bennekou S., Kneuer C., Ossendorp B., Pedersen R., Wolterink G., EFSA (European Food Safety Authority) (2020). Scientific report on the cumulative dietary risk characterisation of pesticides that have chronic effects on the thyroid. EFSA J..

[48] Craig P.S., Dujardin B., Hart A., Hernandez-Jerez A.F., Hougaard Bennekou S., Kneuer C., Ossendorp B., Pedersen R., Wolterink G., EFSA (European Food Safety Authority) (2020). Scientific report on cumulative dietary risk characterisation of pesticides that have acute effects on the nervous system. EFSA J..

