# Supplementary material for: Occurrence of Pesticide Residues in Spanish Honey Measured by QuEChERS Method Followed by Liquid and Gas Chromatography–Tandem Mass Spectrometry
Source: Foods. 2021 Sep 24;10(10):2262. doi: 10.3390/foods10102262 (PMC8534991; doi:10.3390/foods10102262)
Supplement: Supplementary file 1 [file foods-10-02262-s001.zip › foods-1385042-supplementary.pdf]

Table S1. List of Compounds Included in the GC-MS/MS method together with their Retention Times (RT), quantification and confirmation transitions, and collision energies.

Compound	RT (min)	Quantification transition	Collision energy (V)	Confirmation transition	Collision energy (V)
Isoproturon artifact	4.613	146.0 -> 128.0	10	161.0 -> 146.0	10
Dichlorvos	4.675	185.0 -> 93.0	10	185.0 -> 109.0	15
Trichlorfon (artifact)	4.675	185.0 -> 93.0	10	185.0 -> 109.0	15
Disulfoton sulfoxide	5.098	97.0 -> 65.0	15	212.0 -> 125.0	20
2,4,6-Trichlorophenol	5.290	196.0 -> 97.0	35	196.0 -> 132.0	15
Biphenyl	5.443	154.0 -> 115.0	30	154.0 -> 128.0	25
3,5-Dichloroaniline	5.555	161.0 -> 99.0	20	161.0 -> 126.0	15
Mevinphos	5.605	192.0 -> 127.0	10	192.0 -> 164.0	5
Butylate	5.636	156.0 -> 57.0	10	146.0 -> 57.0	5
Chlormephos	5.729	234.0 -> 65.0	25	234.0 -> 121.0	5
Propham	5.782	179.0 -> 93.0	15	179.0 -> 137.0	5
Pebulate	5.817	161.0 -> 128.0	5	203.0 -> 57.0	0
Etridiazole	5.843	211.0 -> 183.0	10	211.0 -> 140.0	25
Phthalimide (Folpet deg)	5.886	147.0 -> 76.0	25	147.0 -> 103.0	5
Tetrahydrophthalimide	5.908	151.0 -> 79.0	20	151.0 -> 122.0	5
Methacrifos	6.056	240.0 -> 180.0	5	208.0 -> 93.0	10
Chloroneb	6.152	191.0 -> 113.0	15	191.0 -> 141.0	10
2-Phenylphenol	6.266	169.0 -> 141.0	15	169.0 -> 115.0	25
Molinate	6.390	187.0 -> 126.0	5	187.0 -> 55.0	20
Heptenophos	6.605	250.0 -> 124.0	5	215.0 -> 89.0	15
DEET (Diethyl-m-toluamide, N,N-)	6.625	190.0 -> 145.0	20	190.0 -> 117.0	25
Chlorfenprop methyl	6.731	196.0 -> 165.0	15	196.0 -> 137.0	25
Propachlor	6.889	176.0 -> 57.0	5	196.0 -> 120.0	10
Tecnazene	6.902	261.0 -> 203.0	10	215.0 -> 179.0	10
Diphenylamine	6.974	169.0 -> 167.0	20	169.0 -> 66.0	25
Ethoprophos	7.014	158.0 -> 97.0	15	200.0 -> 97.0	20
Chlorpropham	7.112	213.0 -> 171.0	5	213.0 -> 127.0	10
Atrazine desisopropyl	7.137	173.0 -> 44.0	10	158.0 -> 91.0	5
Atrazine desethyl	7.216	172.0 -> 94.0	15	172.0 -> 104.0	15
Trifluralin	7.235	306.0 -> 264.0	5	290.0 -> 248.0	5
Benfluralin	7.269	292.0 -> 160.0	25	292.0 -> 264.0	5
Sulfotep	7.364	322.0 -> 146.0	25	322.0 -> 174.0	15
Terbuthylazine-desethyl	7.368	186.0 -> 83.0	20	186.0 -> 145.0	10
Cadusafos	7.415	159.0 -> 97.0	15	213.0 -> 89.0	10
Phorate	7.495	260.0 -> 75.0	10	231.0 -> 129.0	25
Hexachlorocyclohexane-alfa	7.643	217.0 -> 181.0	5	219.0 -> 109.0	5
Thiometon	7.674	88.0 -> 60.0	5	246.0 -> 88.0	0
Prometon	7.783	225.0 -> 168.0	10	225.0 -> 183.0	10

Compound	RT (min)	Quantification transition	Collision energy (V)	Confirmation transition	Collision energy (V)
Hexachlorobenzene	7.788	284.0 -> 214.0	30	284.0 -> 249.0	15
Ethoxyquin	7.793	202.0 -> 174.0	15	202.0 -> 159.0	35
Simazine	7.816	201.0 -> 44.0	15	201.0 -> 138.0	10
Dichloran	7.820	206.0 -> 176.0	10	206.0 -> 148.0	25
Carbofuran	7.833	164.0 -> 103.0	25	164.0 -> 131.0	15
Pentachloroanisole	7.834	280.0 -> 237.0	25	280.0 -> 265.0	10
Atrazine	7.888	215.0 -> 58.0	10	215.0 -> 138.0	15
Propazine	7.946	214.0 -> 172.0	10	229.0 -> 58.0	10
Terbumeton	7.961	225.0 -> 169.0	5	225.0 -> 154.0	15
Clomazone	7.981	204.0 -> 107.0	20	204.0 -> 78.0	30
DMST	8.049	214.0 -> 45.0	10	214.0 -> 106.0	20
Hexachlorocyclohexane-beta	8.054	219.0 -> 183.0	5	219.0 -> 109.0	40
Profluralin	8.097	318.0 -> 199.0	15	318.0 -> 55.0	10
Terbuthylazine	8.118	214.0 -> 104.0	20	214.0 -> 132.0	10
Terbufos	8.151	231.0 -> 129.0	20	231.0 -> 97.0	30
Lindane-gamma	8.152	219.0 -> 183.0	5	219.0 -> 109.0	40
Cyanophos	8.156	243.0 -> 109.0	10	243.0 -> 116.0	8
Quintozene	8.229	295.0 -> 237.0	20	295.0 -> 265.0	5
Fonofos	8.247	246.0 -> 109.0	15	246.0 -> 137.0	5
Diazinon	8.269	304.0 -> 179.0	15	304.0 -> 137.0	40
Pyrimethanil	8.290	198.0 -> 118.0	35	198.0 -> 158.0	20
Phosphamidon I	8.327	264.0 -> 127.0	15	264.0 -> 72.0	10
Tefluthrin	8.405	177.0 -> 127.0	20	177.0 -> 87.0	30
Disulfoton	8.417	88.0 -> 60.0	5	274.0 -> 88.0	10
Paraoxon methyl	8.426	230.0 -> 106.0	15	230.0 -> 136.0	5
Terbacil	8.440	161.0 -> 88.0	20	117.0 -> 76.0	5
Isazophos	8.510	257.0 -> 162.0	5	257.0 -> 161.0	10
Hexachlorocyclohexane-delta	8.523	217.0 -> 181.0	5	219.0 -> 109.0	5
Triallate	8.560	268.0 -> 184.0	20	268.0 -> 226.0	10
Chlorothalonil	8.607	264.0 -> 168.0	25	266.0 -> 133.0	40
Hexachlorocyclohexane-epsilon	8.701	217.0 -> 181.0	5	219.0 -> 109.0	5
Pirimicarb	8.716	238.0 -> 166.0	10	166.0 -> 96.0	15
Fenfluthrin	8.741	181.0 -> 161.0	20	163.0 -> 127.0	5
Formothion	8.765	170.0 -> 93.0	5	170.0 -> 63.0	25
Ethiofencarb	8.776	168.0 -> 107.0	10	168.0 -> 79.0	20
Pirimicarb desmethyl	8.859	224.0 -> 152.0	15	224.0 -> 96.0	20
Pentachloraniline	8.922	263.0 -> 192.0	20	265.0 -> 194.0	20
Phosphamidon II	8.939	264.0 -> 127.0	15	264.0 -> 72.0	10
Dichlofenthion	8.961	279.0 -> 223.0	15	279.0 -> 205.0	30
Propanil	8.966	161.0 -> 99.0	30	161.0 -> 126.0	20
Metribuzin	9.000	198.0 -> 82.0	15	198.0 -> 110.0	10

Compound	RT (min)	Quantification transition	Collision energy (V)	Confirmation transition	Collision energy (V)
Dimethenamide	9.015	230.0 -> 154.1	10	230.0 -> 111.0	25
Malaoxon	9.065	127.0 -> 99.0	5	195.0 -> 125.0	10
Spiroxamine isomer I	9.071	100.0 -> 72.0	5	198.0 -> 126.0	5
Acetochlor	9.074	223.0 -> 132.0	20	223.0 -> 147.0	5
Vinclozolin	9.110	212.0 -> 172.0	15	212.0 -> 145.0	25
Chlorpyrifos-methyl	9.141	286.0 -> 93.0	20	286.0 -> 271.0	15
Parathion Methyl	9.142	263.0 -> 109.0	10	263.0 -> 79.0	30
Fipronil desulfinyl	9.215	333.0 -> 231.0	30	333.0 -> 281.0	15
Isoproturon	9.226	206.0 -> 72.0	20	206.0 -> 146.0	15
Tolclofos Methyl	9.229	265.0 -> 93.0	25	265.0 -> 220.0	25
Ametryn	9.230	227.0 -> 170.0	5	227.0 -> 212.0	10
N-desethylpirimifos methyl	9.239	277.0 -> 166.0	20	277.0 -> 125.0	30
Alachlor	9.247	237.0 -> 160.0	5	269.0 -> 160.0	5
Prometryn	9.276	241.0 -> 184.0	10	241.0 -> 58.0	10
Heptachlor	9.333	272.0 -> 237.0	15	237.0 -> 143.0	35
Fenthion oxon	9.345	262.0 -> 247.0	10	262.0 -> 217.0	15
Fenchlorphos	9.384	285.0 -> 240.0	30	285.0 -> 93.0	25
Prosulfocarb	9.392	251.0 -> 128.0	5	251.0 -> 86.0	10
Fenpropidin	9.454	273.0 -> 98.0	5	273.0 -> 70.0	5
Terbutryn	9.512	241.0 -> 170.0	15	241.0 -> 185.0	10
Spiroxamine isomer II	9.526	100.0 -> 72.0	5	198.0 -> 126.0	5
Pirimiphos Methyl	9.572	290.0 -> 125.0	20	290.0 -> 151.0	15
Fenitrothion	9.585	277.0 -> 109.0	15	260.0 -> 125.0	10
Ethofumesate	9.607	286.0 -> 207.0	5	286.0 -> 161.0	20
Bromacil	9.610	205.0 -> 188.0	15	205.0 -> 162.0	15
Malathion	9.719	173.0 -> 99.0	15	158.0 -> 125.0	10
Dichlofluanid	9.752	224.0 -> 123.0	10	224.0 -> 77.0	45
Metolachlor	9.888	238.0 -> 162.0	10	238.0 -> 133.0	30
Fenpropimorph	9.891	128.0 -> 70.0	10	128.0 -> 110.0	5
Fenthion	9.910	278.0 -> 169.0	15	278.0 -> 125.0	15
Cyanazine	9.918	225.0 -> 189.0	15	225.0 -> 172.0	15
Aldrin	9.934	255.0 -> 220.0	20	263.0 -> 228.0	20
Anthraquinone	9.942	208.0 -> 152.0	20	180.0 -> 152.0	10
Chlorpyrifos	9.949	314.0 -> 258.0	15	314.0 -> 286.0	5
Flufenacet	9.958	211.0 -> 96.0	15	211.0 -> 123.0	5
Parathion Ethyl	9.961	291.0 -> 81.0	40	291.0 -> 109.0	10
4,4'-Dichlorobenzophenone	10.031	250.0 -> 139.0	10	250.0 -> 215.0	5
Chlorthal-dimethyl	10.053	301.0 -> 223.0	25	301.0 -> 273.0	15
Isocarbophos	10.066	230.0 -> 198.0	10	230.0 -> 155.0	20
Butralin	10.214	266.0 -> 174.0	20	266.0 -> 220.0	10
Pirimiphos Ethyl	10.269	318.0 -> 166.0	10	318.0 -> 182.0	10

Compound	RT (min)	Quantification transition	Collision energy (V)	Confirmation transition	Collision energy (V)
Bromophos methyl	10.280	331.0 -> 93.0	25	331.0 -> 286.0	30
Fosthiazate	10.313	195.0 -> 60.0	20	195.0 -> 103.0	5
Isofenphos methyl	10.391	241.0 -> 121.0	15	241.0 -> 199.0	15
Cyprodinil	10.398	224.0 -> 118.0	40	224.0 -> 104.0	25
Chlorphenvinfos I	10.430	267.0 -> 159.0	15	323.0 -> 267.0	10
Isodrin	10.448	262.8 -> 193.0	35	262.8 -> 227.9	20
Pendimethalin	10.512	252.0 -> 162.0	10	252.0 -> 191.0	10
Metazachlor	10.514	209.0 -> 132.0	15	209.0 -> 117.0	35
Terbufos-sulfone	10.543	199.0 -> 97.0	20	264.0 -> 171.0	15
Chlozolinate	10.558	259.0 -> 188.0	10	331.0 -> 259.0	5
Heptachlor-epoxide-A-endo	10.609	289.0 -> 219.0	30	289.0 -> 253.0	10
Heptachlor-epoxide-B-exo	10.613	353.0 -> 263.0	15	353.0 -> 282.0	20
Oxychlordane	10.619	385.0 -> 261.0	15	385.0 -> 285.0	20
Tolylfluanid	10.622	238.0 -> 137.0	15	238.0 -> 91.0	35
Fluopyram	10.630	173.0 -> 95.0	35	223.0 -> 196.0	18
Fipronil	10.642	367.0 -> 213.0	25	367.0 -> 255.0	25
Chlorphenvinfos II	10.644	267.0 -> 159.0	15	323.0 -> 267.0	10
Isofenphos	10.646	213.0 -> 185.0	5	213.0 -> 121.0	10
Mecarbam	10.649	296.0 -> 196.0	10	329.0 -> 131.0	15
Phenthoate	10.719	274.0 -> 121.0	10	274.0 -> 125.0	15
Dinobuton	10.721	211.0 -> 163.0	5	211.0 -> 147.0	10
Quinalphos	10.724	298.0 -> 156.0	20	298.0 -> 190.0	15
Captan	10.734	149.0 -> 70.0	5	151.0 -> 80.0	15
Folpet	10.754	260.0 -> 130.0	15	260.0 -> 102.0	40
Procymidone	10.841	283.0 -> 96.0	10	283.0 -> 67.0	40
Propaphos	10.945	220.0 -> 140.0	10	220.0 -> 125.0	25
Bromophos ethyl	11.016	359.0 -> 303.0	15	359.0 -> 331.0	5
Quinomethionate	11.036	234.0 -> 206.0	10	234.0 -> 148.0	25
Chlordane-trans (gamma)	11.038	373.0 -> 266.0	20	373.0 -> 301.0	10
DDE-o,p'	11.074	318.0 -> 176.0	55	318.0 -> 248.0	15
Tetrachlorvinphos	11.137	329.0 -> 109.0	15	329.0 -> 79.0	35
Disulfoton sulfone	11.145	213.0 -> 153.0	5	213.0 -> 125.0	10
Butachlor	11.192	176.0 -> 146.0	25	237.0 -> 160.0	5
Endosulfan alfa	11.264	241.0 -> 206.0	16	239.0 -> 204.0	16
Picoxystrobin	11.279	335.0 -> 173.0	10	335.0 -> 303.0	10
Chlordane-cis (alpha)	11.295	373.0 -> 266.0	20	373.0 -> 301.0	10
Flutriafol	11.299	219.0 -> 123.0	15	219.0 -> 95.0	35
Fenamiphos	11.303	303.0 -> 154.0	15	303.0 -> 180.0	20
Nonachlor, trans-	11.374	406.8 -> 299.8	15	406.8 -> 108.8	15
Flutolanil	11.376	323.0 -> 173.0	15	323.0 -> 281.0	5
Napropamide	11.395	271.0 -> 72.0	15	271.0 -> 100.0	15

Compound	RT (min)	Quantification transition	Collision energy (V)	Confirmation transition	Collision energy (V)
Iodofenphos	11.460	377.0 -> 157.0	50	377.0 -> 250.0	25
Isoprothiolane	11.480	290.0 -> 118.0	10	290.0 -> 204.0	5
Prothiofos	11.485	309.0 -> 239.0	15	309.0 -> 221.0	30
Profenofos	11.530	339.0 -> 269.0	15	339.0 -> 251.0	35
Fenthion oxon sulfoxide	11.562	262.0 -> 109.0	20	262.0 -> 121.0	35
Fludioxonil	11.568	248.0 -> 127.0	30	248.0 -> 154.0	20
DDE-p,p'	11.617	318.0 -> 176.0	55	318.0 -> 248.0	15
Tricyclazole	11.688	189.0 -> 162.0	10	189.0 -> 135.0	20
Oxyfluorfen	11.697	300.0 -> 223.0	15	361.0 -> 300.0	15
Flamprop-methyl	11.715	276.0 -> 105.0	5	276.0 -> 77.0	40
Dieldrin	11.720	277.0 -> 240.8	5	262.8 -> 227.9	20
Fenthion oxon sulfone	11.725	294.0 -> 104.0	15	294.0 -> 215.0	15
Fipronil sulfone	11.746	383.0 -> 255.0	20	383.0 -> 241.0	10
DDD-o,p'	11.779	235.0 -> 165.0	20	235.0 -> 199.0	15
Bupirimate	11.795	273.0 -> 193.0	5	273.0 -> 108.0	15
Diclobutrazol	11.830	270.0 -> 159.0	15	270.0 -> 201.0	10
Prothioconazol desthio	11.912	186.0 -> 53.0	20	186.0 -> 89.0	15
Fluazifop-butyl	11.963	383.0 -> 282.0	10	383.0 -> 254.0	25
Nitrofen	12.010	283.0 -> 162.0	20	283.0 -> 202.0	15
Chlorfenapyr	12.036	247.0 -> 227.0	15	328.0 -> 247.0	15
Endrin	12.121	242.8 -> 173.0	30	262.8 -> 227.9	20
Chlorobenzilate	12.172	251.0 -> 139.0	15	251.0 -> 111.0	35
Oxadiargyl	12.242	213.0 -> 150.0	10	340.0 -> 213.0	10
Fensulfothion	12.250	293.0 -> 97.0	30	293.0 -> 125.0	10
Fenthion sulfoxide	12.273	279.0 -> 138.0	15	279.0 -> 125.0	25
Endosulfan beta	12.275	241.0 -> 206.0	16	239.0 -> 204.0	16
Flamprop-Isopropyl	12.279	276.0 -> 105.0	5	276.0 -> 77.0	40
Diniconazole	12.314	268.0 -> 232.0	10	268.0 -> 136.0	30
Etaconazole	12.349	245.0 -> 173.0	15	245.0 -> 191.0	10
Fenthion sulfone	12.357	310.0 -> 105.0	15	310.0 -> 109.0	25
DDD-p,p'	12.361	235.0 -> 165.0	20	235.0 -> 199.0	15
DDT-o,p'	12.361	235.0 -> 165.0	20	235.0 -> 199.0	15
Penthiopyrad	12.375	302.0 -> 177.0	15	302.0 -> 152.0	15
Aclonifen	12.396	212.0 -> 182.0	10	264.0 -> 77.0	15
Ethion	12.413	231.0 -> 175.0	10	231.0 -> 129.0	20
Oxadixyl	12.431	163.0 -> 132.0	5	233.0 -> 146.0	10
Nonachlor, cis-	12.483	406.8 -> 299.8	15	406.8 -> 108.8	15
Penflufen	12.575	274.0 -> 141.0	18	274.0 -> 60.0	58
Sulprophos	12.637	322.0 -> 156.0	10	322.0 -> 139.0	10
Triazophos	12.639	257.0 -> 162.0	5	257.0 -> 134.0	25
Ofurace	12.798	232.0 -> 158.0	20	232.0 -> 143.0	35

Compound	RT (min)	Quantification transition	Collision energy (V)	Confirmation transition	Collision energy (V)
Carbophenothion	12.823	342.0 -> 157.0	10	199.0 -> 47.0	20
Benalaxyl	12.859	266.0 -> 148.0	5	234.0 -> 146.0	20
Lenacil	12.958	153.0 -> 136.0	15	153.0 -> 110.0	15
Phosmet oxon	13.008	160.0 -> 51.0	40	160.0 -> 133.0	15
Endosulfan sulfate	13.022	270.0 -> 235.0	15	387.0 -> 289.0	5
Fluopicolide	13.089	347.0 -> 172.0	28	347.0 -> 176.0	15
Dicofol 4,4'-	13.217	251.0 -> 139.0	15	251.0 -> 111.0	35
DDT-p,p'	13.223	235.0 -> 165.0	20	235.0 -> 199.0	15
Methoxychlor deg	13.263	227.0 -> 169.0	25	227.0 -> 141.0	40
Diclofop-methyl	13.266	340.0 -> 253.0	10	340.0 -> 184.0	30
Nuarimol	13.291	235.0 -> 139.0	15	235.0 -> 111.0	40
TPP	13.352	325.0 -> 169.0	20	325.0 -> 231.0	20
Piperonylbutoxide	13.361	176.0 -> 131.0	15	176.0 -> 117.0	20
Resmethrin	13.361	171.0 -> 143.0	5	171.0 -> 128.0	15
Proquinazid	13.370	288.0 -> 245.0	15	288.0 -> 217.0	25
Fluotrimazole	13.482	311.0 -> 165.0	15	311.0 -> 233.0	15
Fenamiphos sulfoxide	13.670	304.0 -> 196.0	5	304.0 -> 122.0	15
Iprodione	13.698	314.0 -> 56.0	20	314.0 -> 245.0	10
Spiromesifen	13.699	272.0 -> 254.0	5	272.0 -> 209.0	10
Fenamiphos sulfone	13.755	320.0 -> 292.0	10	292.0 -> 213.0	10
Pyridaphenthion	13.794	340.0 -> 199.0	5	340.0 -> 203.0	30
Carbosulfan	13.827	160.0 -> 104.0	10	160.0 -> 62.0	20
Tetramethrin	13.861	164.0 -> 77.0	25	164.0 -> 135.0	15
Bifenthrin	13.895	181.0 -> 165.0	25	181.0 -> 115.0	55
Phosmet	13.902	160.0 -> 77.0	20	160.0 -> 133.0	10
Bromopropylate	13.914	341.0 -> 183.0	20	341.0 -> 157.0	45
EPN	13.943	157.0 -> 63.0	10	157.0 -> 110.0	15
Picolinafen	13.943	376.0 -> 238.0	20	376.0 -> 239.0	10
Bifenazate	13.945	258.0 -> 199.0	10	300.0 -> 258.0	5
Methoxychlor	14.015	227.0 -> 169.0	25	227.0 -> 141.0	40
Fenpropathrin	14.027	208.0 -> 181.0	5	265.0 -> 210.0	10
Etoxazol	14.065	300.0 -> 270.0	20	204.0 -> 146.0	30
Fenamidone	14.140	268.0 -> 180.0	20	238.0 -> 103.0	15
Fenazaquin	14.194	160.0 -> 145.0	5	160.0 -> 117.0	20
Bifenox	14.207	341.0 -> 189.0	20	341.0 -> 310.0	10
Metconazol	14.237	250.0 -> 125.0	10	250.0 -> 215.0	15
Fenothrin	14.352	183.0 -> 165.0	10	183.0 -> 153.0	15
Tetradifon	14.411	354.0 -> 159.0	10	354.0 -> 227.0	10
Phosalone	14.568	367.0 -> 182.0	5	367.0 -> 111.0	35
Benthiavalicarb-isopropyl I	14.601	180.0 -> 83.0	30	180.0 -> 127.0	20
Leptophos	14.630	171.0 -> 77.0	15	377.0 -> 269.0	20

Compound	RT (min)	Quantification transition	Collision energy (V)	Confirmation transition	Collision energy (V)
Lambda-Cyhalothrin I	14.668	197.0 -> 161.0	5	208.0 -> 181.0	5
Cyhalofop-butyl	14.676	357.0 -> 229.0	15	357.0 -> 256.0	10
Benthiavalicarb-isopropyl II	14.815	180.0 -> 83.0	30	180.0 -> 127.0	20
Lambda-Cyhalothrin II	14.859	197.0 -> 161.0	5	208.0 -> 181.0	5
Mirex	14.869	272.0 -> 237.0	15	237.0 -> 143.0	30
Acrinathrin I	14.874	289.0 -> 93.0	10	247.0 -> 68.0	30
Acrinathrin II	14.997	289.0 -> 93.0	10	247.0 -> 68.0	30
Fenarimol	15.087	330.0 -> 139.0	5	330.0 -> 111.0	50
Pyrazophos	15.116	265.0 -> 210.0	10	373.0 -> 210.0	15
Azinphos-ethyl	15.208	160.0 -> 77.0	20	160.0 -> 132.0	0
Metrafenone	15.327	393.0 -> 363.0	15	393.0 -> 349.0	20
Isopyrazam	15.328	303.0 -> 159.0	28	303.0 -> 262.0	20
Spirodiclofen	15.642	312.0 -> 109.0	15	312.0 -> 259.0	10
Permethrin	15.689	163.0 -> 127.0	5	165.0 -> 127.0	5
Pyridaben	15.760	309.0 -> 147.0	15	309.0 -> 132.0	40
Fluquinconazole	15.847	340.0 -> 298.0	15	340.0 -> 286.0	25
Coumaphos	15.863	362.0 -> 109.0	15	362.0 -> 226.0	18
Cyfluthrin	16.272	226.0 -> 206.0	15	199.0 -> 170.0	30
Halfenprox	16.536	263.0 -> 129.0	45	263.0 -> 115.0	20
Cypermethrin	16.584	163.0 -> 127.0	5	165.0 -> 127.0	5
Quizalafop ethyl	16.653	372.0 -> 299.0	10	372.0 -> 244.0	25
Flucythrinate I	16.678	199.0 -> 107.0	25	451.0 -> 225.0	5
Pyridalyl	16.795	204.0 -> 148.0	20	204.0 -> 146.0	30
Flucythrinate II	16.862	199.0 -> 107.0	25	451.0 -> 225.0	5
Fenvalerate	17.388	167.0 -> 125.0	5	225.0 -> 147.0	15
Fluvalinate tau	17.574	250.0 -> 55.0	15	250.0 -> 200.0	15
Esfenvalerate	17.583	167.0 -> 125.0	5	225.0 -> 147.0	15
Deltamethrin I	17.897	251.0 -> 172.0	5	172.0 -> 93.0	10
Deltamethrin II	18.104	251.0 -> 172.0	5	172.0 -> 93.0	10

Table S2. List of Compounds Included in the LC-MS/MS method together with their Retention Times (RT), quantification and confirmation transitions, capillary voltage, and collision energies.

Compound	RT (min)	Polarity	Capillary voltage (V)	Quantification transition	Collision energy	Confirmation transition	Collision energy
Methamidophos	3.22	Positive	44	142 > 94	10.5	142 > 125	11.5
Pymetrozine	3.22	Positive	52	218 > 105	15	218 > 218	6.5
Formetanate	3.25	Positive	48	222 > 165	13	222 > 222	5
Acephate	3.28	Positive	30	201 > 143	9.5	201 > 184	5

Compound	RT (min)	Polarity	Capillary voltage (V)	Quantification transition	Collision energy	Confirmation transition	Collision energy
Omethoate	3.34	Positive	36	214 > 125	19	214 > 183	9.5
Aldicarb sulfoxide	3.37	Positive	32	207 > 132	4.5	207 > 89	10
Propamocarb	3.38	Positive	48	189 > 102	13.5	189 > 144	9.5
Oxamyl	3.41	Positive	30	237 > 72	5.5	237 > 90	5
Aldicarb sulfone	3.41	Positive	30	240 > 86	4.5	240 > 75.9	9
Oxydemeton-methyl	3.48	Positive	32	247 > 109	23.5	247 > 169	12
Demeton-S-methylsulfone	3.52	Positive	48	263 > 121	12	263 > 169	13.5
Methomyl	3.55	Positive	32	163 > 88	5	163 > 106	6
Thiamethoxam	3.56	Positive	52	292 > 211	9.5	292 > 132	15.5
Monocrotophos	3.57	Positive	32	224 > 193	6.5	224 > 127	12
Carbendazim	3.59	Positive	68	192 > 132	25.5	192 > 160	14
Flonicamid	3.60	Negative	40	228 > 81	10	228 > 228	5
Spirotetramat enol-glucoside	3.62	Positive	60	464 > 302	12	464 > 216.1	42
Dimethylphenil-N-methyllformanidina DMPF	3.65	Positive	44	163 > 122	11.5	163 > 107	19.5
Metiocarb sulfone	3.69	Positive	30	275 > 122	14.5	275 > 107	7
Metiocarb sulfoxide	3.73	Positive	30	242 > 185	4.5	242 > 122.1	23
Imidacloprid	3.73	Positive	52	256 > 175	16.5	256 > 209	13.5
Thiabendazole	3.75	Positive	68	202 > 131	27	202 > 175	19.5
Vamidothion	3.83	Positive	30	288 > 118	18.5	288 > 146	8.5
Acetamiprid	3.86	Positive	32	223 > 90	29	223 > 126	17
3-OH carbofuran	3.87	Positive	52	238 > 163	12.5	238 > 219.9	4.5
Trichlorfon	3.89	Positive	30	274 > 109	15.5	274 > 257	6.5
Clothianidin	3.78	Positive	40	250 > 169	11	250 > 132	13
Dimethoate	3.95	Positive	30	230 > 125	19	230 > 171	14
Thiacloprid	4.02	Positive	52	253 > 90	30	253 > 126	17
Sulfoxaflor	4.03	Positive	60	278 > 174	8	278 > 278	5
Cymoxanil	4.13	Positive	30	199 > 128	6	199 > 111.2	16
Ethirimol	4.42	Positive	68	210 > 140	19.5	210 > 98.1	25.5
Aldicarb	4.44	Positive	30	208 > 89	10.5	208 > 116	5
Spirotetramat mono hydroxy	4.67	Positive	60	304 > 254	15	304 > 211.1	22
Thiophanate-methyl	4.70	Positive	44	343 > 151	15.5	343 > 311	8
Propoxur	4.84	Positive	30	210 > 93	24	210 > 111	12
Carofurano	4.86	Positive	40	222 > 137	17	222 > 165	9.5
Thiodicarb	5.05	Positive	30	355 > 88	8.5	355 > 108	11.5
Carbaryl	5.13	Positive	30	202 > 117	19.5	202 > 145	7
Carboxin	5.13	Positive	36	236 > 143	11	236 > 93	27.5
Spirotetramat cis enol	5.34	Positive	60	302 > 216	30	302 > 270	20
Imazalil	5.46	Positive	76	297 > 159	18	297 > 201	15.5
Metalaxyl	5.86	Positive	44	280 > 220	12.5	280 > 248	8.5
Spirotetramat cis keto hydroxy	6.09	Positive	60	318 > 214	25	318 > 300.3	4
Chlorantraniliprole	6.26	Positive	52	484 > 285.6	10.5	484 > 484	5

Compound	RT (min)	Polarity	Capillary voltage (V)	Quantification transition			Collision energy	Confirmation transition			Collision energy
Methidathion	6.27	Positive	48	303	>	85	15.5	303	>	145	7
Azinphos methyl	6.37	Positive	30	318	>	132	11.5	318	>	160	6.5
Azoxystrobin	6.67	Positive	30	404	>	344	21	404	>	372	15
Diethofencarb	6.87	Positive	32	268	>	180	16	268	>	226	8.5
Dimethomorph	7.07; 7.79	Positive	44	388	>	301	15	388	>	165	29.5
Linuron	7.18	Positive	40	249	>	160	14.5	249	>	182	11.5
Methiocarb	7.34	Positive	32	226	>	121	15.5	226	>	169	8
Triflusulfuron methyl	7.36	Positive	44	493	>	264	18.5	493.4	>	461	9.5
Mandipropamid	7.46	Positive	52	413	>	328.3	11.5	413	>	356.3	7
Fludioxonil	7.57	Negative	-72	247	>	126	30.5	247	>	180	26
Boscalid	7.46	Positive	72	343	>	307	15.5	343	>	271	27.5
Malathion	8.16	Positive	105	331	>	99	18	331	>	127	10
Paclobutrazol	7.68	Positive	48	295	>	70	13	295	>	125	30.5
Metoxifenocide	7.77	Positive	30	369	>	149	15.5	369	>	313	7
Fluxapyroxad	7.90	Negative	-60	380	>	248	14	380	>	131	12
Propyzamide	7.97	Positive	48	256	>	173	21	256	>	190	13
Cyproconazole	8.01; 8.54	Positive	64	292	>	70	13	292	>	125	26.5
Triadimefon	7.397	Positive	60	294	>	69	18	294	>	197	14
Fenpyrazamine	8.08	Positive	60	332	>	70	15	332	>	272	15
Myclobutanil	8.16	Positive	68	289	>	70	9.5	289	>	125	27.5
Bromuconazole	8.23;9.58	Positive	89	378	>	159	17.5	377.9	>	161	18.5
Mepanipyrim	8.40	Positive	68	224	>	224	9.5	224	>	105.5	27.5
Iprovalicarb	8.41	Positive	32	321	>	119	14	321	>	203	7
Spirotetramat	8.43	Positive	60	374	>	330	8	374	>	302	10
Triadimenol	8.56	Positive	30	296	>	70	7	296	>	99	11.5
Fenhexamid	8.65	Positive	64	302	>	55	26.5	302	>	97	18.5
Triticonazole	8.74	Positive	60	318	>	70	12	318	>	125	30.5
Diclofluanid	8.79	Positive	68	333	>	224	9.5	333	>	123	22.5
Tetraconazole	8.86	Positive	72	373	>	70	22.5	373	>	159.3	24.5
Epoxiconazole	8.88	Positive	40	330	>	121	18	330	>	101	37.5
Cyazofamid	8.97	Positive	50	325	>	108	9.5	325	>	261	6.5
Rotenone	9.13	Positive	40	395	>	192	21.5	395	>	213	20.5
Fenbuconazole	9.17	Positive	68	337	>	70	13,5	337	>	125	22.5
Tebufenocide	9.26	Positive	32	353	>	133	16,5	353	>	297	7
Flusilazole	9.32	Positive	44	316	>	165	24.5	316	>	247	15.5
Diflubenzuron	9.37	Positive	30	311	>	158	8	311	>	141	25
Fenoxycarb	9.39	Positive	30	302	>	88	15	302	>	116	7
Kresoxim-methyl	9.55	Positive	40	314	>	116	9.5	314	>	206	6
Bixafen	9.58	Positive	60	414	>	394	8	414	>	266	15
Cyflufenamid	9.72;10.31	Positive	50	413	>	295	12	413	>	203	25
Tolyfluanide	9.97	Positive	30	364	>	238	13	364	>	137	23.5
Penconazole	9.75	Positive	64	284	>	70	10.5	284	>	159	27.5
Tebuconazole	9.88	Positive	60	308	>	70	13	308	>	125	32

Compound	RT (min)	Polarity	Capillary voltage (V)	Quantification transition			Collision energy	Confirmation transition			Collision energy
TPP	10.03	Positive	76	327	>	152	16.5	327	>	215	20
Prochloraz	10.09	Positive	32	376	>	70	18	376	>	308	10.5
Zoxamide	10.11	Positive	55	336	>	187	17	336	>	159	36.5
Propiconazole	10.11	Positive	76	342	>	69	13	342	>	159	26
Famoxadone	10.16	Positive	40	392	>	238	16	392	>	331	7.5
Pyraclostrobin	10.21	Positive	30	388	>	194	10	388	>	163	21.5
Hexaconazole	10.32	Positive	68	314	>	70	11	314	>	159	26
Spinosyn A	10.41	Positive	40	733	>	142	25.5	733	>	733	8.5
Triflumuron	10.41	Negative	-48	357	>	154	11	357	>	175.8	21
Clofentezine	10.42	Positive	36	303	>	138	11.5	303	>	102.1	30
Bitertanol	10.47	Positive	42	338	>	70	5	338	>	99	10.5
Pencycuron	10.57	Positive	44	329	>	125	21	329	>	329	6
Dodine	10.59	Positive	44	228	>	60	16.5	228	>	186	15.5
Ametoctradin	10.78	Positive	60	276	>	176	30	276	>	149	38
Tebufenpyrad	11.65	Positive	88	334	>	96	26	334	>	185	21
Tolclofos-methyl	10.93	Positive	43	301	>	269	12.5	300.7	>	125	22
Indoxacarb	10.92	Positive	44	528	>	150	23.5	528	>	218	22
Trifloxystrobin	10.92	Positive	30	409	>	186	16	409	>	206	12
Difenoconazole	10.89	Positive	52	407	>	251	19	407	>	337	10
Spinosyn D	11.07	Positive	32	747	>	142	26	747	>	98	9.5
Spinetoram I	11.07	Positive	50	749	>	142	28	748.5	>	748.5	5
Metaflumizone	11.07;11.83	Positive	60	507	>	178	24	507	>	287	24
Benfuracarb	11.28	Positive	48	411	>	195	22	411	>	252.1	12
Novaluron	11.29;12.73	Positive	50	493	>	158	12	493	>	141	22
Tebufenpyrad	11.65	Positive	88	334	>	117	25	334	>	145	21
Spinetoran II	11.68	Positive	65	761	>	142	24	760.5	>	98	50
Pyriproxyfen	12.06	Positive	44	322	>	96	11.5	322	>	185	21.5
Lufenuron	12.09	Negative	-30	510	>	326	16.5	510	>	339	10
Emamectin benzoate B1a	12.10	Positive	88	887	>	158	29.5	887	>	887	8
Teflubenzuron	12.14	Negative	-44	379	>	339	8	379	>	359	5
Quinoxyfen	12.20	Positive	92	308	>	162.1	28.5	308	>	197	25.5
Hexythiazox	12.26	Positive	48	353	>	228	13	353	>	168	23
Buprofezin	12.52	Positive	70	306	>	116	14	306	>	201	8
Spiromesifen	12.59	Positive	60	371.5	>	273.4	9.5	371.5	>	255	21
Propargite	12.58	Positive	32	368	>	175	15	368	>	231	9.5
Flufenoxuron	12.73	Negative	-40	487	>	156	14	487	>	411	11
Fenpyroximate	12.85	Positive	48	422	>	138	13	422	>	366	23
Amitraz	12.94	Positive	44	294	>	163	13	294.1	>	122	24
Clorflurazon	13.18	Positive	44	540	>	383	17	540	>	158.1	19.5
Abamectin	14.07	Positive	44	891	>	305	25.5	890.5	>	567	11.5
Etofenprox	14.86	Positive	44	394	>	135	23.5	394	>	177	13
Fenbutatin oxide	16.41	Positive	104	519	>	197	45	519	>	288.8	31
